# Supplementary material for: Expression of the Endoplasmic Reticulum Stress Marker GRP78 in the Normal Retina and Retinal Degeneration Induced by Blue LED Stimuli in Mice
Source: Cells. 2021 Apr 23;10(5):995. doi: 10.3390/cells10050995 (PMC8145904; doi:10.3390/cells10050995)
Supplement: Supplementary file 1 [file cells-10-00995-s001.zip › cells-1178694-supplementary.pdf]

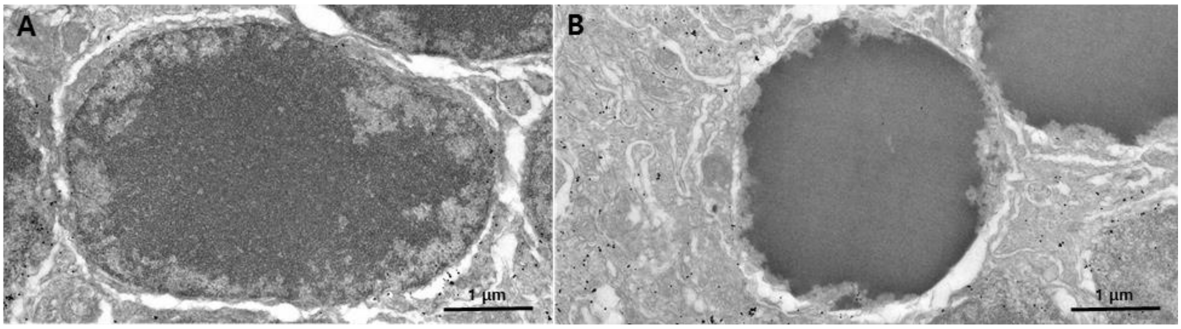

Scheme 1. Subcellular localization of the GRP78 in the ONL of the normal retina and blue LED–induced RD retina. Immuno-gold labeled GRP78 was detected inter-cellular space which was mainly consisted by Müller glia processes in both normal (**A**), and apoptotic photoreceptor (**B**).

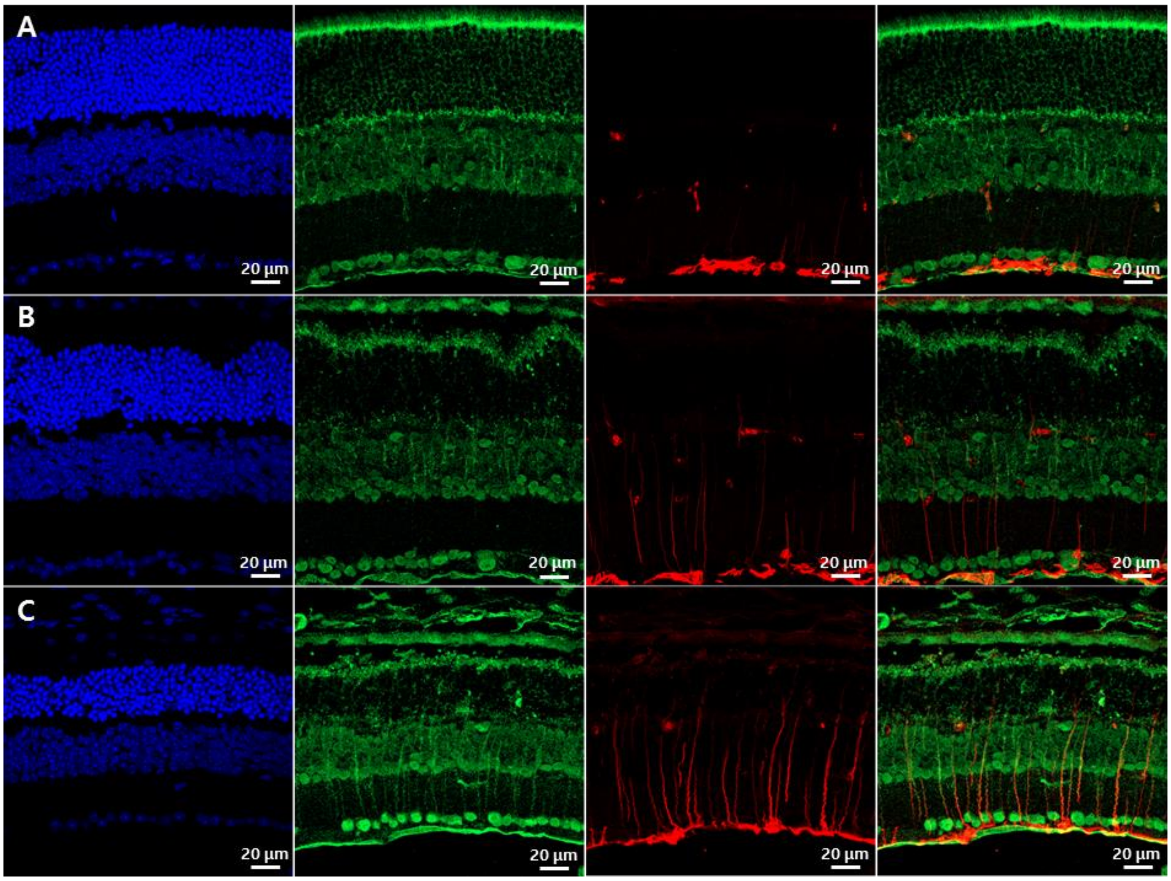

Scheme 2. Expression of the calreticulin in blue LED–induced RD. DAPI (blue), calreticulin (green), GFAP (red) were labeled in the normal retina and 24 hrs, 72 hrs after LED exposure retina. (**A**) In the normal retina, calreticulin were mainly detected in the segment layer ONL, ONL and GCL, and GFAP was rarely labeled in the IPL to ONL. (**B**) After 24 hrs from light exposure, change of the calreticulin was not prominent while GFAP were increased in the IPL. (**C**) After 72 hrs, calreticulin in the ONL, INL and IPL was prominently increased, and GFAP was increased from INL to ONL more than 24 hrs group. Increased calreticulin and GFAP were definitely co-localized in the 72 hrs retina.
